# Uric acid is independently associated with interleukin‐1β levels in tear fluid of hyperuricemia and gout patients

**DOI:** 10.1002/iid3.805

**Published:** 2023-03-17

**Authors:** Mian Wu, Xingna Hu, Ting Lu, Chenxiao Liu, Honghong Lu

**Affiliations:** ^1^ Department of Endocrinology and Metabolism, The Affiliated Suzhou Hospital of Nanjing Medical University Suzhou Municipal Hospital Suzhou China

**Keywords:** gout, hyperuricemia, inflammation, interleukin‐1beta, tears

## Abstract

**Objectives:**

To explore pro‐inflammatory cytokines status in the tear fluid of patients with hyperuricemia and gout and its association with uric acid level.

**Methods:**

A total of 58 control subjects, 58 hyperuricemia patients including 40 asymptomatic hyperuricemia and 18 gout participants were recruited in this study. For tear analysis, each patient's tears were collected using capillary action microcaps after stimulation. Tear uric acid levels were measured using chemiluminescence. Tear and serum interleukin‐1beta (IL‐1β) and tumor necrosis factor‐alpha (TNF‐α) levels were measured using enzyme‐linked immunosorbent assay. The correlation of serum and tear uric acid levels with IL‐1β and TNF‐α were determined.

**Results:**

Tear uric acid levels were significantly higher in hyperuricemia group (98.2 ± 51.5 vs. 42.7 ± 24.0 µmol/L, *p* < .001) than in controls group. IL‐1β concentrations were significantly higher in hyperuricemia eyes compared to control eyes (210.2 ± 113.9 vs. 142.6 ± 29.8 pg/mL, *p* < .001). Multiple linear regression analysis showed that tear uric acid levels were independently positively associated with tear IL‐1β concentrations (*B* = 0.192, *p* < .001). However, no significant correlations were found between serum or tear uric acid and TNF‐α level. Moreover, there were no statistically differences of tear IL‐1β and TNF‐α levels between the asymptomatic hyperuricemia and gout groups.

**Conclusions:**

Tear uric acid levels were higher in patients with hyperuricemia and gout than in controls. There was a significant positive correlation between tear uric acid value and tear IL‐1β level, implying an interaction between hyperuricemia and ocular inflammation responses.

## Graphical abstract

Tear urate levels were higher in patients with hyperuricemia and gout than in controls. There was a significant positive correlation between tear uric acid and tear IL‐1β level.

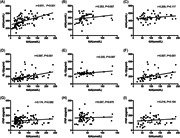

## 1 INTRODUCTION

Uric acid is the end product of purine catabolism. Either overproduction or underexcretion of uric acid can lead to hyperuricemia. It is estimated that up to 21% of the general population and 25% of hospitalized patients have asymptomatic hyperuricemia.^1^ Hyperuricemia is the most crucial risk factor for gout development; moreover, it is also associated with a variety of comorbidities, including hypertension, diabetes, chronic kidney disease, and coronary artery disease.^2^ However, the impact of hyperuricemia on ocular abnormalities remained poorly understood.

The eye is a particularly vulnerable organ, susceptible to vascular abnormalities, metabolic disturbances, and inflammation. For instance, diabetic retinopathy is a leading cause of vision impairment caused by metabolic stress, glucose‐mediated microvascular damage, and inflammation.^3^ In hyperurcemia and gout patients, urate crystal deposition has been observed in almost all ocular and adnexal locations.^4^ Furthermore, in addition to lower temperatures and pH level or pH gradient between plasma and tissue, ocular structures have relatively poor solvent ability, indicating that they are predisposed to tophi deposition.^5^ Crystal‐induced inflammation of the affected tissue due to urate deposition is responsible for the clinical presentations. Therefore, it is necessary to study the changes in ocular inflammation in hyperuricemia and gout patients.

Tear fluid is a heterogeneous solution containing mainly proteins, lipids, mucins, and electrolytes, responsible for regulating the physiology of human eye. The complex composition of tears can be altered due to eye inflammations.^6^ Research has indicated that pro‐inflammatory factors such as interleukin (IL)‐1β, IL‐17, and IL‐8, NOD‐like receptor protein 3 (NLRP3) inflammasome, tumor necrosis factor‐alpha (TNF‐α), and anti‐inflammatory factors such as IL‐10, IL‐37 have all been implicated monosodium urate (MSU)‐induced gout inflammatory process.^7^ However, cytokines in the tears of hyperuricemia and gout patients remained unknown.

Therefore, this study aims to compare the difference of uric acid and pro‐inflammatory cytokine levels in tears between healthy controls and hyperuricemia patients, and explore the correlation between tear uric acid and pro‐inflammatory cytokines levels.

## 2 MATERIALS AND METHODS

### 2.1 Study population

Participants were recruited from our inpatient department at the Affiliated Suzhou Hospital of Nanjing Medical University between June 2020 and October 2021. The study was conducted in accordance with the Declaration of Helsinki and was approved by the ethics committee at the Affiliated Suzhou Hospital of Nanjing Medical University (KL901220). All participants gave written informed consent.

Inclusion criteria for hyperuricemia subjects were (i) age between 18 and 80 years, (ii) serum uric acid level ≥ 420 µmol/L, and (iii) history of hyperuricemia or gout flare. Diagnosis of gout was based on the 2015 American College of Rheumatology/European League Against Rheumatism diagnostic criteria.^8^ Exclusion criteria for all subjects were as follows: (i) any ophthalmological condition such as infection or allergic conjunctivitis, (ii) severe liver or kidney impairment (alanine aminotransferase [ALT] or aspartate aminotransferase [AST] greater than three times the normal value or estimated glomerular filtration rate (eGFR) lower than 30 mL/min/1.73 m^2^), (iii) pregnant or breastfeeding women, (iv) any other severe medical, neurological or psychiatric condition, and (v) gout patients during acute flare and patients receiving regular uric acid‐lowering therapy, nonsteroidal anti‐inflammatory drugs (NSAIDs), colchicine, corticosteroids, and diuretics within 1 month.

In total, 68 healthy controls and 65 hyperuricemia patients were assessed for eligibility. Of these, 10 controls and 7 patients were excluded due to insufficient amount of tear fluid sampled, leaving 58 patients and 58 hyperuricemia patients included for final analysis.

### 2.2 Tear collection

Tear fluid sampling was conducted between 7:00 a.m. and 9:00 p.m. in a fasting condition. Reflex tears were collected as previously described.^9^ After sneeze reflex was stimulated by gently inserting a sterile cotton bud into the nasal passages to induce tear production, tear collection took place for approximately 1 min to enable sufficient volume to be obtained. Participants tilted their head toward the side of the collection while looking in the opposite direction. The microcapillary tube rested in the lateral tear meniscus. Participants were allowed to blink during the procedure. The sample was expelled from the capillary tube into a siliconized polypropylene microcentrifuge tube of 0.25 mL capacity and placed on ice until processing. After collection, samples were centrifuged at 4000 rpm for 20 min at 4°C to remove cellular debris. The supernatants were collected and stored at −80°C.

### 2.3 Tear fluid analyses

Tear uric acid level were determined using chemiluminescence (Nanjing Jiancheng Bioengineering Institute). To detect tear IL‐1β and TNF‐α concentrations, the tear fluids were diluted 20 times with phosphate buffered saline and detected using a commercial enzyme‐linked immunosorbent assay kit, according to the user manual (R&D systems).

### 2.4 Clinical and laboratory assessments

The clinical data of the subjects were recorded, including height, weight, systolic blood pressure, and diastolic blood pressure. The body mass index (BMI) was calculated as weight (kg)/height (m^2^). Venous blood samples were collected in the morning after an overnight fast to measure ALT, AST, serum uric acid, urea nitrogen, creatinine, blood glucose, cholesterol, and triglycerides. The estimated glomerular filtration rate (GFR) was calculated from the four‐variable modification of diet in renal disease equation: estimated GFR (mL/min/1.73 m^2^) = 175 × (serum creatinine [μmol/L]/88.4)^–1.154^ × (age [years])^–0.203^ × (0.742 if female) × (1.21 if Black). All subjects underwent standard clinical and laboratory evaluations.

### 2.5 Statistics

Continuous variables were represented as mean ± standard deviation, whereas categorical variables were displayed as numbers with percentages. Student's *t* test was used to compare means for normally distributed variables, and the *χ* ^2^ test was used to compare frequencies between groups. Spearman's *ρ* was used to test for correlations. Variables that were significantly associated with tear uric acid were defined as candidate factors for linear regression analysis. A multivariable linear regression model using the stepwise likelihood ratio method was fitted with candidate factors, with entry probability of 0.05 and removal of 0.10. analysis of covariance was used to test differences in tear uric acid between asymptomatic hyperuricemia and gout patients after adjusting for serum uric acid level. All tests were two‐tailed, and *p* < .05 were considered statistically significant. Statistical analyses were performed using the SPSS software package, version 21.0.

## 3 RESULTS

### 3.1 General characteristics of subjects in control and hyperuricemia group

The patient demographics are summarized in Table 1. The mean age of control and hyperuricemia subjects was 49.2 and 50.7 years, respectively. 72.4% and 82.8% of participants were males in control and hyperuricemia group. Compared with controls, patients in the hyperuricemia group were more obese, had higher levels of systolic blood pressure and fasting blood glucose, and had worse liver and kidney functions. In all patients, we did not observe significant uric acid deposition in the cornea.

**Table 1:** Comparison of clinical characteristics between healthy controls and hyperuricemia patients.

Variable	Control	HUA	*p* Value
*N* (male)	58 (42)	58 (48)	.219
Age (year)	48.2 ± 13.0	50.7 ± 17.5	.395
BMI (kg/m^2^)	23.5 ± 3.0	26.1 ± 4.3	<.001
SBP (mmHg)	127.3 ± 16.8	136.6 ± 22.1	.012
DBP (mmHg)	77.0 ± 11.5	79.1 ± 13.0	.372
ALT (U/L)	24.6 ± 23.1	36.6 ± 36.1	.012
AST (U/L)	23.1 ± 11.7	27.0 ± 16.1	.372
BUN (mmol/L)	4.9 ± 1.4	6.5 ± 3.7	.002
Cr (umol/L)	70.8 ± 14.1	91.7 ± 33.9	<.001
eGFR (mL/min/1.73 m^2^)	106.4 ± 22.6	89.5 ± 34.2	<.001
FPG (mmol/L)	5.9 ± 1.9	6.7 ± 2.1	.044
TC (mmol/L)	4.8 ± 0.8	4.5 ± 1.1	.110
TG (mmol/L)	1.5 ± 1.1	1.9 ± 1.3	.052
sUA (umol/L)	292.9 ± 67.2	479.7 ± 89.1	<.001
Gout (%)	0	31.0	‐

*Note*: Categorical variables are shown as numbers, and continuous variables are presented as means ± standard deviations.

Abbreviations: BUN, blood urea nitrogen; Cr, creatinine; DBP, diastolic blood pressure; eGFR, estimated glomerular filtration rate; FPG, fasting plasma glucose; HUA, hyperuricemia; SBP, systolic blood pressure; sUA, serum uric acid; TC, total cholesterol; TG, triglycerides; WHR, waist:hip ratio.

### 3.2 Quantitative analysis of uric acid level, pro‐inflammatory cytokines in tear fluid

**Figure 1:**
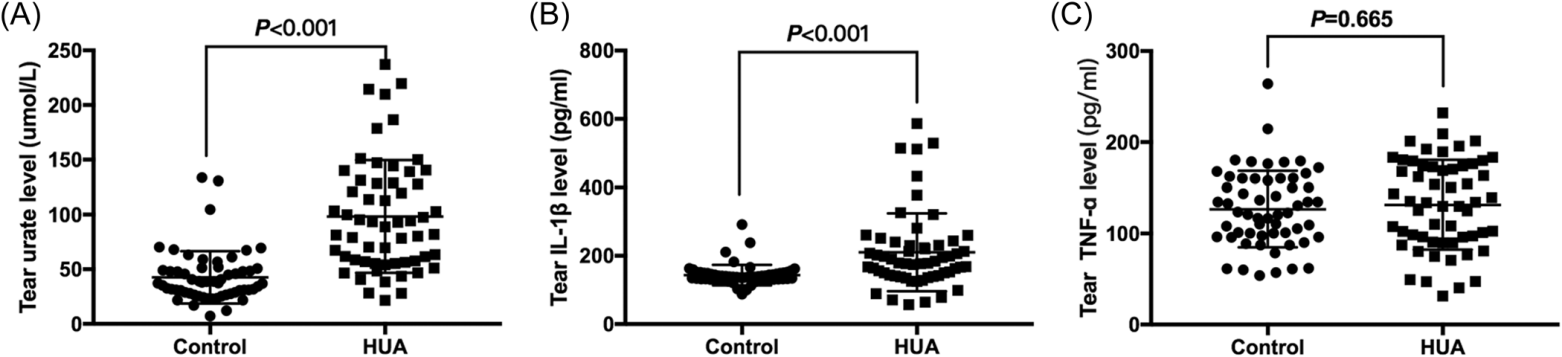
Comparison of tear uric acid and pro‐inflammatory cytokines levels between control and hyperuricemia groups. (A) Comparison of tear uric acid (A), tear IL‐1β (B) and tear TNF‐α (C) between control and HUA groups. HUA, hyperuricemia; tIL‐1β, tear interleukin‐1beta; tTNF‐α, tear tumor necrosis factor‐α; tUA, tear uric acid.

As illustrated in Figure 1A, tear uric acid levels in hyperuricemia group were significantly higher than control group (98.2 ± 51.5 vs. 42.7 ± 24.0 µmol/L, *p* < .001). Pro‐inflammatory cytokines including IL‐1β and TNF‐α were evaluated in tear fluid. The results illustrated that tear IL‐1β level was significantly higher in hyperuricemia group than in healthy control group (210.2 ± 113.9 vs. 143.6 ± 29.8 pg/mL, *p* < .001) (Figure 1B), whereas no significant difference existed in tear TNF‐α levels between control and hyperuricemia groups (130.5 ± 50.5 vs. 134.7 ± 54.8 pg/mL, *p* = .665) (Figure 1C).

### 3.3 Correlation between uric acid and other clinical variables

**Figure 2:**
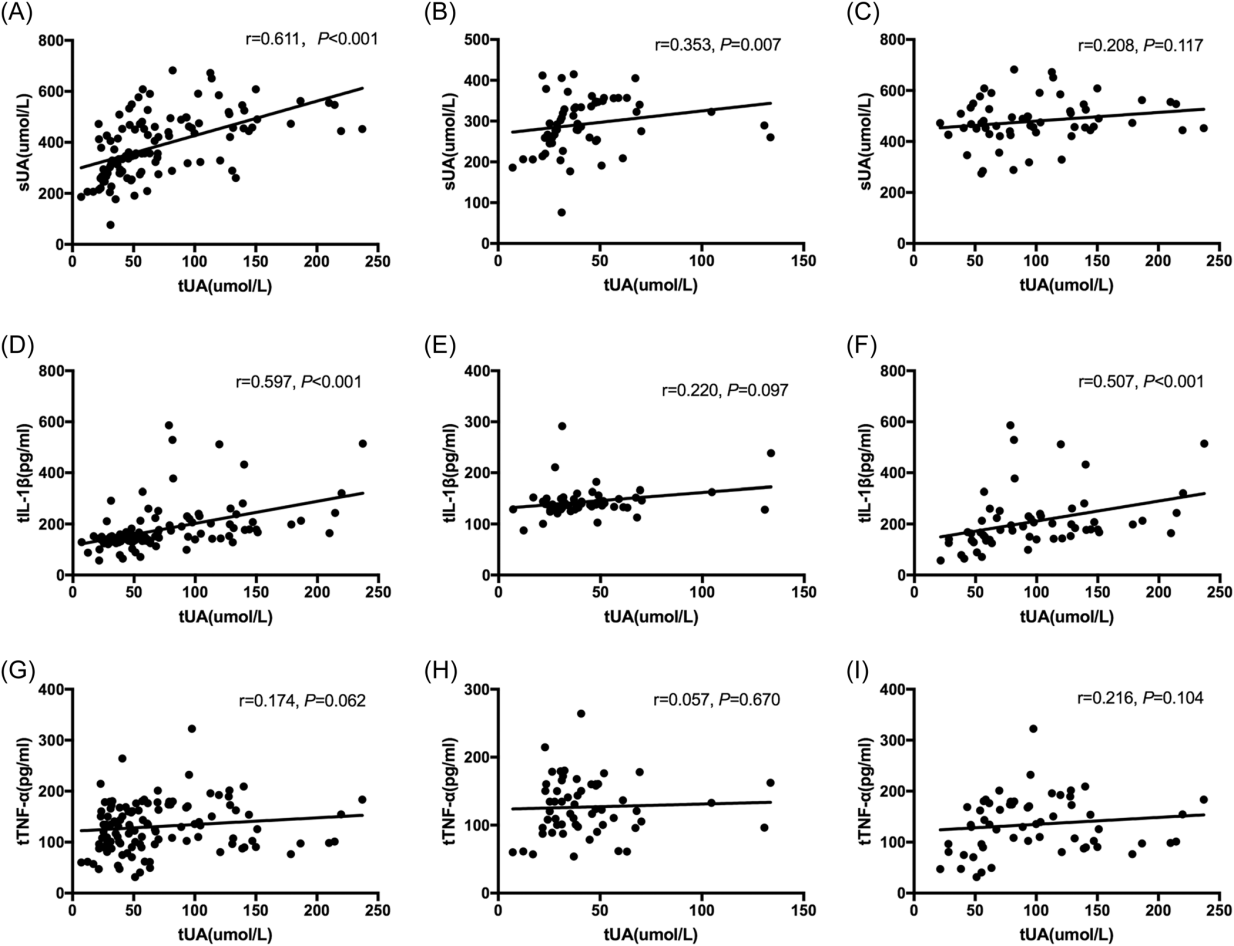
Spearman correlations between tear uric acid and serum uric acid, tear IL‐1β or tear TNF‐α concentrations. Correlation between tear uric acid and serum uric acid of total population (A), control group (B) or HUA group (C). Correlation between tear uric acid and tear IL‐1β of total population (D), control group (E) or HUA group (F). Correlation between tear uric acid and tear TNF‐α of total population (G), control group (H), or HUA group (I). HUA, hyperuricemia; IL‐1β, interleukin‐1beta; TNF‐α, tear tumor necrosis factor‐α.

Tear uric acid level revealed a positively association with BMI, Cr, and TG levels, serum uric acid level was significantly positively correlated with BMI, SBP, ALT, Cr, and TG (Table 2). As shown in Figure 2A–C, tear uric acid level was significantly positively correlated with serum uric acid level in total participants and control group, but not in hyperuricemia group. Moreover, there were positive associations between tear uric acid level and tear IL‐1β level in total participants and the hyperuricemia group, but not in the control group (Figure 2D–F). However, no statistically significant correlations were observed between tear uric acid and tear TNF‐α level in either total participants, healthy controls, or hyperuricemia patients (Figure 2G–I).

**Table 2:** Factors correlated with tear and serum uric acid levels.

	Tear uric acid		Serum uric acid	
	*r*	*p* Value	*r*	*p* Value
Age	.151	.106	−.040	.668
BMI	.303	.001**	.349	<.001***
SBP	.015	.871	.225	.015*
DBP	−.098	.293	.127	.176
ALT	.088	.347	.280	.002**
AST	.047	.614	.149	.109
BUN	.175	.060	.120	.200
Cr	.273	.003**	.480	<.001***
eGFR	−.286	.002**	−.290	.002**
FPG	.148	.114	.128	.172
TC	−.084	.371	−.084	.370
TG	.227	<.001***	.372	<.001***
tIL‐1β	.597	<.001***	.355	.003**
tTNF‐α	.174	.062	.051	.590

Abbreviations: BUN, blood urea nitrogen; Cr, creatinine; DBP, diastolic blood pressure; eGFR, estimated glomerular filtration rate; FPG, fasting plasma glucose; HUA, hyperuricemia; SBP, systolic blood pressure; sUA, serum uric acid; TC, total cholesterol; TG, triglycerides; tIL‐1β, tear interleukin‐1beta; tTNF‐α, tear tumor necrosis factor‐α; WHR, waist:hip ratio.

* *p* < .05

** *p* < .01

*** *p* < .001.

### 3.4 Tear uric acid was independently associated with tear IL‐1β concentration

Variables significantly related with tear uric acid in Spearman correlation analysis including BMI, SBP, ALT, Cr, TG, sUA, and tear IL‐1β were included in multivariable linear regression analysis. As indicated in Table 3, tear uric acid was independently associated with serum uric acid (*B*, 0.177; 95% confidence interval [CI], 0.118–0.256; *p* < .001) and tear IL‐1β (*B*, 0.192; 95% CI, 0.111–0.273; *p* < .001).

**Table 3:** Multivariable linear regression of factors associated with tear uric acid level.

	*B*	*p* Value	95% CI	
			Upper limit	Lower limit
sUA	0.177	<.001	0.118	0.256
tIL‐1β	0.192	<.001	0.111	0.273

Abbreviations: 95% CI, 95% confidence interval; sUA, serum uric acid; tIL‐1β, tear tear interleukin‐1beta.

### 3.5 Tear uric acid and cytokine levels between asymptomatic hyperuricemia patients and acute gout patients

Eighteen patients in the hyperuricemia group experienced acute gout attacks before and were in intermittent period at the time of enrollment. Therefore, we divided the hyperuricemia subjects into the asymptomatic hyperuricemia group and gout group. As indicated in Table 4, serum uric acid level was slightly higher in the asymptomatic hyperuricemia group than that in gout group; and tear uric acid concentration was significant higher in asymptomatic hyperuricemia group than in gout patients. However, after adjusting for serum uric acid level, the difference of tear uric acid level between two groups was no longer significant (*p* = .076). Furthermore, there were no statistical differences of tear IL‐1β and TNF‐α levels between asymptomatic hyperuricemia group and gout group, suggesting that ocular inflammation has been overexpressed during the asymptomatic hyperuricemia period.

**Table 4:** Comparison of uric acid level and pro‐inflammatory cytokines between asymotomatic HUA and gout patients.

	Asymptomatic HUA	Gout	*p* Value
*N* (male)	40 (32)	18 (17)	.249
sUA (umol/L)	497.8 ± 66.1	439.3 ± 118.9	.063
tUA (umol/L)	109.3 ± 54.5	73.5 ± 33.7	.013
tIL‐1β (pg/mL)	222.3 ± 115.2	183.1 ± 109.3	.229
tTNF‐α (pg/mL)	138.6 ± 57.7	116.1 ± 47.9	.429

Abbreviations: sUA, serum uric acid; tIL‐1β, tear interleukin‐1beta; tTNF‐α, tear tumor necrosis factor‐α; tUA, tear uric acid.

## 4 DISCUSSION

The current study demonstrated that hyperuricemia patients had significantly higher tear uric acid levels than healthy controls. Tear IL‐1β concentration was elevated in the hyperuricemia group, and significantly positively linked to tear uric acid level, implying an interaction between hyperuricemia and inflammation responses. However, no significant differences existed in tear IL‐1β or TNF‐α levels between asymptomatic hyperuricemia and acute gout flare groups, suggesting that as early as the asymptomatic hyperuricemia period, eye inflammation may exist.

This study discovered that the average uric acid concentration in tear fluid of control subjects and hyperuricemia patients was 42.7 and 98.2 μmol/L, respectively, significantly lower than those in serum. Consistent with our study, a former study found that mean concentrations of uric acid were lower in tear than in serum (119 vs. 345 μmol/L).^10^ At the same time, we found that the level of tear uric acid in this study was lower than that reported in other literatures, which may be related to the different methods of tear collection and uric acid detection techniques used.

Hyperuricemia can cause ocular surface abnormalities, such as tophi deposition, subconjunctival transparent vesicles and hemorrhage, and vascular changes.^11^ Conjunctival hyperemia and subconjunctival hemorrhage exacerbated by purine intake are two of the more common manifestations.^4^ Other ocular and adnexal structures may be affected by urate deposition and associated inflammation, with potentially sight‐threatening consequences.^4^

Multiple studies have implicated the relationship between serum uric acid and IL‐1β release.^12^ IL‐1β plays a key role in the pathogenesis and in the screen for specific treatments of various inflammatory and degenerative eye diseases.^13^ For instance, studies using a uveitis rat model reported a significant upregulation of IL‐1β and TNF‐α gene expression in the stage of active intraocular inflammation.^14^, ^15^ IL‐1 antagonists have been successfully used to treat uveitis in monogenic autoinflammatory diseases such as Blau syndrome and cryopyridine‐associated periodic syndrome or in complex polygenic autoinflammatory diseases such as Behçet's disease.^16^, ^17^ Likewise, treatment with the IL‐1 receptor blocker anakinra has also been shown to be successful in scleritis and episcleritis in different rheumatic conditions.^18^, ^19^ Thus, IL‐1β concentration may be considered a potential indicator and treat target of hyperuricemia‐associated ocular disorders. The current study indicated that hyperuricemia patients had significantly higher tear IL‐1β levels. A positive association was observed between tear IL‐1β and serum/tear uric acid levels, implying that elevated tear uric acid level may be involved in the development of eye inflammation related to hyperuricemia.

The actual hyperuricemia cut‐off is principally based on the saturation point of uric acid. Recent study has shown that the negative impact of cardiovascular disease could occur at lower levels.^20^ Another study indicated that the optimal cut‐off values for serum uric acid to identify metabolic syndrome were 6.3 mg/dL in men and 4.9 mg/dL in women.^21^ These results emphasizes the importance of treat‐to‐target for approach of uric acid lowering treatment in patients with hyperuricemia. However, there are no data about the uric acid cut‐off associated to the risk for uric acid‐induced ocular damage, which needs further research in the future.

However, compared with patients with asymptomatic hyperuricemia and acute gout attacks, there was no significant difference in the level of IL‐1β. Typically, gout is recognized as painful arthritis due to urate deposition, which can be treated intermittently with anti‐inflammatory drugs and some medical associations recommend initiating uric acid‐lowering therapies only when repeated flares occur.^22^ Systemic deposition of urate and resulting chronic inflammation may be the potential link to the frequent comorbidities associated with gout.^23^ Our results suggest that as early as asymptomatic hyperuricemia period, eye inflammation may exist.

Several limitations should be considered. First, the sample size in this study was small, especially in some of the subgroup analyses. Second, our cross‐sectional study design precludes commenting on persistence or change in tear uric acid and cytokines concentration, and MSU disposition features in hyperuricemia and gout patients over time. Third, the ophthalmic manifestations of gout are rare but diverse. In this study, we did not find significant corneal urate deposition. However, direct clinical data were not available to assess ocular or extraocular manifestations. Fourth, tear IL‐1β and TNF‐α was explored in this study. Many cytokines such as IL‐1Ra, IL‐33, IL‐37, IL‐38, and IL‐6 levels were not assessed.

In conclusion, we have demonstrated that upregulated uric acid and IL‐1β levels in the tear fluid of hyperuricemia and gout patients. Upregulated tear uric acid and IL‐1β levels identified in the eyes of hyperuricemia patients imply IL‐1β‐mediated inflammation may be a plausible mechanism underlying hyperuricemia and gout‐associated ocular symptoms. Moreover, asymptomatic hyperuricemia population present the same level of IL‐1β and TNF‐α compared with patients with gout flare, highlighting the importance of physicians paying special attention to asymptomatic hyperuricemia. More prospective studies with a larger sample size are required, however, to confirm the current study's findings and explore the association of elevated cytokines and ocular symptoms in hyperuricemia and gout patients.

## AUTHOR CONTRIBUTIONS

**Mian Wu**: Data curation; funding acquisition; investigation; software; writing—original draft; writing—review and editing. **Xingna Hu**: Data curation; formal analysis; methodology; software; writing—original draft. **Ting Lu**: Data curation; formal analysis; methodology. **Chenxiao Liu**: Data curation; funding acquisition; visualization. **Honghong Lu**: Conceptualization; data curation; project administration; resources; supervision; validation; writing—review and editing.

## CONFLICT OF INTEREST STATEMENT

The authors declare no conflict of interest.

## Supporting information

Supplementary information.

## Data Availability

All the data used to support the findings of this study are available from the corresponding author upon reasonable request.

## References

[1] George C , Minter DA . Hyperuricemia. StatPearls; 2021. 29083565

[2] Yip K , Cohen RE , Pillinger MH . Asymptomatic hyperuricemia: is it really asymptomatic? Curr Opin Rheumatol. 2020;32(1):71‐79. 31688126 10.1097/BOR.0000000000000679

[3] Forrester JV , Kuffova L , Delibegovic M . The role of inflammation in diabetic retinopathy. Front Immunol. 2020;11:583687. 33240272 10.3389/fimmu.2020.583687 PMC7677305

[4] Ao J , Goldblatt F , Casson RJ . Review of the ophthalmic manifestations of gout and uric acid crystal deposition. Clin Experiment Ophthalmol. 2017;45(1):73‐80. 27010973 10.1111/ceo.12749

[5] Coassin M , Piovanetti O , Stark WJ , Green WR . Urate deposition in the iris and anterior chamber. Ophthalmology. 2006;113(3):462‐465. 16513461 10.1016/j.ophtha.2005.11.012

[6] Capaccio A , Sasso A , Rusciano G . Raman analysis of tear fluid alteration following contact lense use. Sensors. 2019;19(15):3392. 31382386 10.3390/s19153392 PMC6695878

[7] Wu M , Tian Y , Wang Q , Guo C . Gout: a disease involved with complicated immunoinflammatory responses: a narrative review. Clin Rheumatol. 2020;39(10):2849‐2859. 32382830 10.1007/s10067-020-05090-8

[8] Neogi T , Jansen TLTA , Dalbeth N , et al. 2015 gout classification criteria: an American College of Rheumatology/European League Against Rheumatism collaborative initiative. Ann Rheum Dis. 2015;74(10):1789‐1798. 26359487 10.1136/annrheumdis-2015-208237 PMC4602275

[9] Markoulli M , Papas E , Petznick A , Holden B . Validation of the flush method as an alternative to basal or reflex tear collection. Curr Eye Res. 2011;36(3):198‐207. 21275520 10.3109/02713683.2010.542867

[10] Horwath‐Winter J , Kirchengast S , Meinitzer A , Wachswender C , Faschinger C , Schmut O . Determination of uric acid concentrations in human tear fluid, aqueous humour and serum. Acta Ophthalmol. 2009;87(2):188‐192. 18547277 10.1111/j.1755-3768.2008.01215.x

[11] Lin J , Zhao GQ , Che CY , Yang SS , Wang Q , Li CG . Characteristics of ocular abnormalities in gout patients. Int J Ophthalmol. 2013;6(3):307‐311. 23826523 10.3980/j.issn.2222-3959.2013.03.09 PMC3693010

[12] Klück V , Liu R , Joosten LAB . The role of interleukin‐1 family members in hyperuricemia and gout. Joint Bone Spine. 2021;88(2):105092. 33129923 10.1016/j.jbspin.2020.105092

[13] Fabiani C , Sota J , Tosi GM , et al. The emerging role of interleukin (IL)‐1 in the pathogenesis and treatment of inflammatory and degenerative eye diseases. Clin Rheumatol. 2017;36(10):2307‐2318. 28032234 10.1007/s10067-016-3527-z

[14] Yoshida M , Yoshimura N , Hangai M , Tanihara H , Honda Y . Interleukin‐1 alpha, interleukin‐1 beta, and tumor necrosis factor gene expression in endotoxin‐induced uveitis. Invest Ophthalmol Visual Sci. 1994;35(3):1107‐1113. 8125721

[15] Planck SR , Huang XN , Robertson JE , Rosenbaum JT . Cytokine mRNA levels in rat ocular tissues after systemic endotoxin treatment. Invest Ophthalmol Visual Sci. 1994;35(3):924‐930. 8125755

[16] Simonini G , Xu Z , Caputo R , et al. Clinical and transcriptional response to the long‐acting interleukin‐1 blocker canakinumab in Blau syndrome‐related uveitis. Arthritis Rheum. 2013;65(2):513‐518. 23124805 10.1002/art.37776 PMC3684148

[17] Gül A , Tugal‐Tutkun I , Dinarello CA , et al. Interleukin‐1β‐regulating antibody XOMA 052 (gevokizumab) in the treatment of acute exacerbations of resistant uveitis of Behçet's disease: an open‐label pilot study. Ann Rheum Dis. 2012;71(4):563‐566. 22084392 10.1136/annrheumdis-2011-155143

[18] Knickelbein JE , Tucker WR , Bhatt N , et al. Gevokizumab in the treatment of autoimmune non‐necrotizing anterior scleritis: results of a phase I/II clinical trial. Am J Ophthalmol. 2016;172:104‐110. 27663070 10.1016/j.ajo.2016.09.017 PMC5121021

[19] Botsios C , Sfriso P , Ostuni PA , Todesco S , Punzi L . Efficacy of the IL‐1 receptor antagonist, anakinra, for the treatment of diffuse anterior scleritis in rheumatoid arthritis. Report of two cases. Rheumatology. 2007;46(6):1042‐1043. 17449489 10.1093/rheumatology/kem052

[20] Maloberti A , Giannattasio C , Bombelli M , et al. Hyperuricemia and risk of cardiovascular outcomes: the experience of the URRAH (Uric Acid Right for Heart Health) project. High Blood Press Cardiovasc Prev. 2020;27(2):121‐128. 32157643 10.1007/s40292-020-00368-z

[21] Zhang M , Gao Y , Wang X , Chang H , Huang G . Serum uric acid and appropriate cutoff value for prediction of metabolic syndrome among Chinese adults. J Clin Biochem Nutr. 2013;52(1):38‐42. 23341696 10.3164/jcbn.12-65 PMC3541417

[22] Qaseem A , Harris RP , Forciea MA , Clinical Guidelines Committee of the American College of P , Denberg TD , et al. Management of acute and recurrent gout: A clinical practice guideline from the American College of Physicians. Ann Intern Med. 2017;166(1):58‐68. 27802508 10.7326/M16-0570

[23] Khanna P , Johnson RJ , Marder B , LaMoreaux B , Kumar A . Systemic urate deposition: an unrecognized complication of gout? J Clin Med. 2020;9(10):3204. 33023045 10.3390/jcm9103204 PMC7600842

